# How to measure wisdom: content, reliability, and validity of five measures

**DOI:** 10.3389/fpsyg.2013.00405

**Published:** 2013-07-12

**Authors:** Judith Glück, Susanne König, Katja Naschenweng, Uwe Redzanowski, Lara Dorner, Irene Straßer, Wolfgang Wiedermann

**Affiliations:** Department of Developmental Psychology, Alpen-Adria-Universität KlagenfurtKlagenfurt, Austria

**Keywords:** measurement of wisdom, self-assessed wisdom scale, three-dimensional wisdom scale, adult self-transcendence inventory, berlin wisdom paradigm, reliability, validity

## Abstract

Wisdom is a field of growing interest both inside and outside academic psychology, and researchers are increasingly interested in using measures of wisdom in their work. However, wisdom is a highly complex construct, and its various operationalizations are based on quite different definitions. Which measure a researcher chooses for a particular research project may have a strong influence on the results. This study compares four well-established measures of wisdom—the Self-Assessed Wisdom Scale (Webster, 2003, 2007), the Three-Dimensional Wisdom Scale (Ardelt, 2003), the Adult Self-Transcendence Inventory (Levenson et al., 2005), and the Berlin Wisdom Paradigm (Baltes and Smith, 1990; Baltes and Staudinger, 2000)—with respect to content, reliability, factorial structure, and construct validity (relationships to wisdom nomination, interview-based wisdom ratings, and correlates of wisdom). The sample consisted of 47 wisdom nominees and 123 control participants. While none of the measures performed “better” than the others by absolute standards, recommendations are given for researchers to select the most suitable measure for their substantive interests. In addition, a “Brief Wisdom Screening Scale” is introduced that contains those 20 items from the three self-report scales that were most highly correlated with the common factor across the scales.

## Introduction

**Figure 1:**
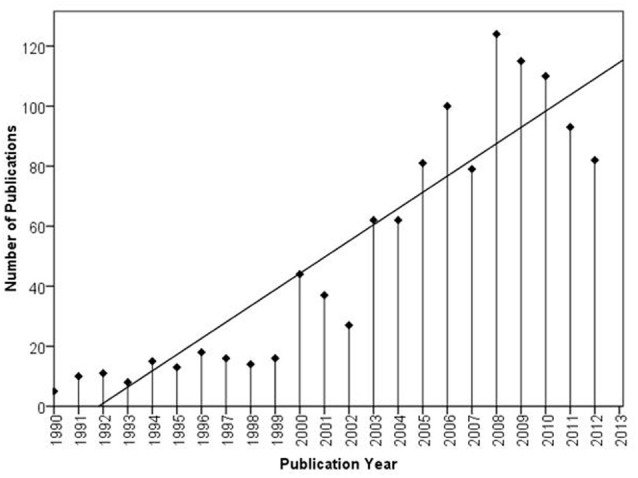
**Number of publications with “wisdom” as subject term in PsycInfo since 1990**.

Wisdom is a field of growing interest both inside and outside academic psychology. Over the last two decades, psychological wisdom research has grown steeply in terms of quantity (see Figure 1) as well as quality and sophistication of operationalizations and research designs (review in Staudinger and Glück, 2011; for recent innovations see, e.g., König and Glück, submitted; Kross and Grossmann, 2012; Grossmann et al., 2013; Thomas and Kunzmann, submitted). Beyond genuine wisdom research, the concept of wisdom is increasingly being applied to relevant fields such as clinical psychology and psychotherapy (e.g., Germer and Siegel, 2012), decision-making (e.g., Yaniv and Choshen-Hillel, 2012), leadership (e.g., Kilburg, 2012), and education (e.g., Sternberg, 2010). Therefore, an increasing number of researchers from other fields are interested in using measures of wisdom in their work. However, it is not easy to get an overview of the field and select the measure that is most optimally suited for a particular study. A number of measures are available, each representing a particular theory of wisdom, and the conceptual differences between the theories are large. Here, we have conceptually analyzed and empirically investigated four popular measures of wisdom: How do they differ in content, how are they interrelated, and how do their correlate structures differ? As we and others have found (e.g., Taylor et al., 2011), correlations among wisdom measures are surprisingly low, so the choice of a particular measure may strongly influence study results. While no measure is “better” than the others by absolute standards, we try to derive specific recommendations for researchers. We also introduce a “Brief Wisdom Screening Scale” that contains those 20 items that had the highest correlations to a general wisdom factor.

Measures of wisdom have been grouped in two different but overlapping ways (e.g., Staudinger and Glück, 2011). First, there are self-report measures and performance-based measures. The three self-report measures in this study were Ardelt's (2003) Three-Dimensional Wisdom Scale (3D-WS), Webster's (2003) Self-Assessed Wisdom Scale (SAWS), and Levenson and colleagues' Adult Self-Transcendence Inventory (ASTI; Levenson et al., 2005). Performance-based measures assess wisdom from a person's verbal responses to wisdom-requiring problems. They include the Berlin wisdom paradigm (BWP; overview in Baltes and Staudinger, 2000), which was used in the current study, and the Bremen wisdom paradigm (Mickler and Staudinger, 2008).

The second distinction is between measures of “personal wisdom” and measures of “general wisdom” (Staudinger et al., 2005; Staudinger and Glück, 2011). Personal wisdom is obtained through personal experiences and insights concerning one's own life, whereas general wisdom concerns human life and the world in general and is not necessarily related to personal experience. The self-report scales listed above and the Bremen wisdom paradigm (Mickler and Staudinger, 2008) measure personal wisdom, whereas the Berlin wisdom paradigm is the only well-established assessment of general wisdom (Staudinger and Glück, 2011).

In this study, we have administered the 3D-WS, ASTI, SAWS, and BWP to a sample of wisdom nominees and a general-population control group. In the following, we first look at the *content validity* of the measures as represented by the authors' definitions of wisdom and the actual scale items. Then, we analyze the *reliability, structural relationships*, and *construct validity* of the measures. At the end, we introduce the new “Brief Wisdom Screening Scale.” Rather than to decide which measure is “best,” our main goal is to derive recommendations for researchers depending on their specific interests.

## Materials and methods

This research was approved by the ethics committee of the German Psychological Society. Participants were treated in accordance with the Declaration of Helsinki. All participants gave their informed written consent to participate in the study.

### Participants

The sample included 47 wisdom nominees and 123 control participants. The nominees were recruited through newspaper and radio calls in the Austrian province of Carinthia, asking anyone who knew a particularly wise person to nominate that person to the project team. Self-nominations were not accepted. A total of 82 people were nominated, and 47 of them agreed to participate. Most control participants were recruited through a commercially available random sample of about 1600 Carinthians; a few were included through personal contacts of students and colleagues.

The wisdom nominees were 23 women and 24 men aged 26–92 years (*M* = 60.9, *SD* = 16.3). Of the nominees, 57.4% were married or living with a partner, 38.3% had a university degree, and 42.6% were retired. The “*total control group*” included 67 women and 56 men aged 19–95 years (*M* = 54.1, *SD* = 15.8); 67.5% were married or living with a partner, only 13.0% had a university degree, and 37.4% were retired. To reduce costs, interviews, including the BWP, were transcribed only for a subgroup of 47 control participants parallel in age and gender to the nominees. This “*parallel control group*” included 23 women and 24 men aged 26–84 years (*M* = 60.0, *SD* = 15.1), of whom 63.9% were married or living with a partner, 8.5% had a university degree, and 57.4% were retired.

The wisdom nominees were higher in education than both the total control group, χ^2^(2, *N* = 143) = 10.31, *p* = 0.006, and the parallel control group, χ^2^(2, *N* = 94) = 11.66, *p* = 0.003. They also scored higher in vocabulary than both the total, *t*(161) = 3.09, *p* = 0.002, and the parallel control group, *t*(88) = 2.37, *p* = 0.020, but not in inductive reasoning (total control group: *t*[162] = 0.24, *p* = 0.808; parallel control group: *t*[88] = 0.98, *p* = 0.332). Because of these group differences, we controlled education and vocabulary in group comparisons and analyzed relationships of the wisdom measures to education and vocabulary.

### Measures

#### Wisdom measures

The SAWS (Webster, 2003, 2007; Taylor et al., 2011) measures five components of wisdom: openness, emotional regulation, humor, critical life experience, and reminiscence and reflectiveness. It consists of 40 items presented with a 6-point Likert scale from “strongly disagree” to “strongly agree.” Webster (2007) reported a Cronbach's alpha of 0.90. The SAWS is positively related to ego integrity, forgiveness, personal well-being, generativity, and positive psychosocial values, and negatively to attachment avoidance. It is unrelated to education and age, but women score higher than men (Webster, 2003, 2007, 2010; Taylor et al., 2011). For the current study, the scale items were translated into German and back-translated by native German and English speakers to optimize the translation.

Monika Ardelt's 3D-WS (Ardelt, 2000, 2003, 2011) defines wisdom as the combination of a cognitive (14 items), reflective (12 items), and affective dimension (13 items). Of the items, 24 are presented with a 5-point response scale from “definitely true of myself” to “not true of myself,” and 15 are presented with a 5-point Likert scale from “strongly agree” to “strongly disagree.” The German 3D-WS was provided by the scale author, who is a native speaker of German. Ardelt (2003) reported Cronbach's alphas from 0.71 to 0.85 for the three dimensions and a 10-month test-retest correlation of 0.85. The 3D-WS is positively related to mastery, purpose in life, forgiveness, and well-being, and negatively to depression, economic pressure, death avoidance, and fear of death. It is unrelated to gender, negatively correlated to age, and positively correlated to education (Ardelt, 2003, 2011).

The ASTI (Levenson et al., 2005) measures wisdom as self-transcendence. While the items of the original ASTI all referred to self-perceived changes over time, we used a revised version, provided by the scale authors, that is presented with a four-point scale from “disagree strongly” to “agree strongly.” Of its 35 items, 10 refer to alienation and 25 to self-transcendence; only the self-transcendence items were analyzed here. The scale was translated into German and back-translated by native German and English speakers. Levenson et al. (2005) reported a Cronbach's alpha of 0.75 for the 10 items measuring self-transcendence in the original ASTI. The original ASTI was positively related to openness to experience, extraversion, meditation practice, and egalitarianism, and negatively related to neuroticism, vertical individualism, and immature love (Le and Levenson, 2005; Levenson et al., 2005).

The *Berlin Wisdom Paradigm (BWP)* is a performance measure of wisdom-related knowledge (see, e.g., Baltes and Smith, 1990; Baltes and Staudinger, 2000). In the current study, we used the “life-review problem:” *In reflecting over their life, people sometimes realize that they have not achieved what they had once wanted to achieve. What could a person consider and do in such a situation?* Participants' spoken responses to difficult life problems are transcribed and rated according to five criteria: factual knowledge, procedural knowledge, life-span contextualism, value relativism, and recognition and management of uncertainty. BWP performance is correlated to life experience, openness to experience, personal growth, intelligence, creativity, thinking styles, affective involvement, and self- and other-enhancing values (Staudinger et al., 1997, 1998; Kunzmann and Baltes, 2003).

#### Correlates of wisdom

Seven potential correlates of wisdom were included in this study. *Self-efficacy* was measured using the German original version of the Generalized Self-Efficacy Scale (Schwarzer and Jerusalem, 1995), which consists of ten items measuring general optimistic expectations about one's own competency. *Emotional competence* was measured using the German-language Emotional Competence Questionnaire (Freudenthaler and Neubauer, 2005; see also Freudenthaler et al., 2008). It is a 34-item self-report scale that measures self-related and other-related perception and regulation of emotions. To measure *empathy*, we used a German translation of the Empathic Concern subscale (7 items) of the Interpersonal Reactivity Index (Davis, 1983). *Openness* was measured using the 12 items of the Openness to Experience subscale of the German NEO-FFI (Borkenau and Ostendorf, 1993). *Psychological well-being* was assessed by a German short version of the Ryff Scales of Psychological Well-Being (Ryff and Keyes, 1995) that was previously used in studies with the BWP (Staudinger and Baltes, 1996; Glück and Baltes, 2006). As an indicator of fluid intelligence, we measured *inductive reasoning* using a 15-item short form of the Matrices subtest of the German CFT-20-R (Weiss, 2008). For crystallized intelligence, we assessed participants' *vocabulary* with the German “Mehrfachwahl-Wortschatz-Intelligenztest” (multiple-choice vocabulary intelligence test; Lehrl, 2005), which consists of 37 items.

#### Interview data

As described in detail elsewhere (König and Glück, submitted; Glück et al. submitted), participants were interviewed by trained project members about a highly difficult life experience and an important past conflict. The interview included a free narrative of the event and structured questions concerning how the participant—and, in the conflict, the opponent—had felt at the time, how they had dealt with the event, how they thought about it today, and whether they had learned something from it.

### Procedure

Most participants came to the laboratory for two interview sessions; a few older participants were interviewed at home or a place of their choice. Participants were also requested to fill out materials before and between the two sessions. Before the first session, they filled out the 3D-WS, the self-efficacy scale, the NEO openness scale, the Ryff scales, the SAWS, and the Life Story Matrix (Glück and Bluck, 2007). After the first session, they completed the emotional-competence scales, the ASTI, and a few measures irrelevant to the present topic. The empathy scale was later mailed to the participants together with several measures of gratitude (see König and Glück, submitted).

In Session 1, participants were first presented with introductory tasks for the BWP (following the manual by Staudinger et al., 1994) and then with the BWP “life review” task. They were then interviewed about the most difficult and best event listed in their Life Story Matrix. The second interview session, about 2 weeks later, included the measures of inductive reasoning and vocabulary, the interview about a difficult conflict, and free accounts of their most important life lessons and insights. On average, each interview session took about 1.5 h, with a range of 1–4 h. Participants received € 70 (about US$ 100) for completing both sessions.

### Ratings

For cost reasons, only the interviews with the wisdom nominees and the parallel control group were transcribed and rated. The BWP responses were rated by 10 students (two for each wisdom criterion), who were trained as described in the BWP manual (Staudinger et al., 1994) and received a payment of € 300 (about $ 400). They rated each protocol on a 7-point scale ranging from “very little correspondence to an ideal response” (1) to “very strong correspondence to an ideal response” (7). Overall wisdom ratings for the interview transcripts about difficult life events and conflicts were obtained from a different panel of 28 student coders who received course and practical-training credit. They rated each protocol on a 4-point scale from “no indications of wisdom” (0) to “extraordinary level of wisdom” (3).

## Results

In the following, we present analyses concerning the content validity (including face validity), reliability, structural relationships, and construct validity of the four wisdom measures.

### Content validity

The four measures of wisdom differ both in how wisdom is defined and how those definitions are operationalized. Therefore, we first present an analysis of the wisdom definition and the concrete item content of each measure. Table 1 shows sample items for the subscales of the self-report measures and idealized quotations representing the criteria of the BWP.

**Table 1:** **Sample items from the wisdom measures (BWP “sample items” are idealized quotations from study participants)**.

**Subscale/Criterion**	**Sample item**
**SAWS**	
Critical life experience (8 items, α = 0.82)	I have overcome many painful events in my life.
	I have experienced many moral dilemmas.
Emotional regulation (8 items, α = 0.71)	I am good at identifying subtle emotions within myself.
	It is easy for me to adjust my emotions to the situation at hand.
Reminiscence and reflectiveness (8 items, α = 0.88)	I often think about my personal past.
	Remembering my earlier days helps me gain insight into important life matters.
Humor (8 items, α = 0.84)	I can chuckle at personal embarrassments.
	I try and find a humorous side when coping with a major life transition.
Openness (8 items, α = 0.72)	I like being around persons whose views are strongly different from mine.
	I'm very curious about other religious and/or philosophical belief systems.
**3D-WS**	
Reflective dimension (12 items, α = 0.77)	Things often go wrong for me by no fault of my own. *(reversed)*
	I always try to look at all sides of a problem.
	Before criticizing somebody, I try to imagine how I would feel if I were in their place.
Affective dimension (13 items, α = 0.61)	I am annoyed by unhappy people who just feel sorry for themselves. *(reversed)*
	Sometimes I feel a real compassion for everyone.
	I can be comfortable with all kinds of people.
Cognitive dimension (14 items, α = 0.74)	You can classify almost all people as either honest or crooked. *(reversed)*
	I try to anticipate and avoid situations where there is a likely chance I will have to think in depth about something. *(reversed)*
	It is better not to know too much about things that cannot be changed. *(reversed)*
**ASTI**	
Self-transcendence (25 items, α = 0.83)	My peace of mind is not easily upset.
	I feel that my individual life is a part of a greater whole.
	Different parts of me are often at cross purposes. *(reversed)*
	Whatever I do to others, I do to myself.
**BWP**	
Factual knowledge (Inter-rater *r* = 0.68)	“ ‘Reflecting upon their lives’ can mean very different things, depending on why the person is looking back and what situation they are in. Old people often look back to find meaning or some patterns in their life story, while younger people … ”
Procedural knowledge (Inter-rater *r* = 0.72)	“When someone feels they have not achieved what they wanted to achieve, perhaps they should instead look for things they have achieved, that is, shift their focus. They might also examine the reasons why some things did not work out, because there are often benefits even in losses …”
Life-span contextualism (Inter-rater *r* = 0.47)	“This is obviously dependent on the age and life phase of the person, and also on their life situation and the chances they have to change something. A young person may have more opportunities than a middle-aged or old person to adjust their goals and means. An old person may have a very different perspective.“
Value relativism (Inter-rater *r* = 0.61)	“What one person views as highly important goals may be totally unimportant for another, and this often leads to conflicts. It is important to be aware of such differences, for example when a married couple has different views on how to deal with work and family. There is never one person who is ‘right’ in such conflicts.”
Uncertainty (Inter-rater *r* = 0.53)	“Well, now I've been talking so much but I really don't know how any of this would work out in a real situation. There are so many things that can happen in someone's life, and people are so different, it is very hard to give good advice even to people you know well.”

The SAWS (Webster, 2003, 2007; Taylor et al., 2011) is based on a definition of wisdom as “the *competence* in, *intention* to, and *application* of, *critical* life experiences to facilitate the *optimal development* of *self* and *others*” (Webster, 2007, p. 164; italics by original author). *Openness* concerns “alternate views, information, and potential solution strategies” (Webster, 2007, p. 166) but also one's inner experiences. The scale items refer to interest and willingness to engage in music, books, art, and food, new things in general, and perspectives different from one's own. *Emotional regulation* refers to “an exquisite sensitivity to the gross distinctions, subtle nuances, and complex blends of the full range of human affect” (Webster, 2007, p. 166), which includes the ability and willingness to recognize, embrace, and constructively employ emotions. Notably, one of the eight items in the scale, “It seems I have a talent for reading other people's emotions,” is about the emotions of others, while all others concern the participant's own emotions. *Humor* refers to being able to recognize irony and to use humor to reduce stress and bond with others (Webster, 2007, p. 167). Two of the eight scale items refer to using humor with others, the others are about laughing about one's own flaws or finding something amusing in difficult situations. *Critical Life Experience* refers to important personal experiences “which are morally ambiguous, multifaceted, and fraught with unknown outcomes” (Webster, 2007, p. 167) but also positive events that may serve as resources. With the exception of one item (“I've learned valuable life lessons from others”), the scale items all pertain to having had a particular kind of experience, mostly negative or difficult. *Reminiscence and Reflectiveness* refers to an evaluative and integrative reflection of one's past and present that helps one to deal with future difficulties. The scale items refer to the frequency with which participants reminisce and to the use of reminiscence to deal with the present.

The 3D-WS (Ardelt, 2000, 2003, 2011) measures three components of wisdom. The *reflective dimension* is considered necessary for developing the other two dimensions, as an individual needs to be willing to look “at phenomena and events from many different perspectives to develop self-awareness and self-insight” (Ardelt, 2003, p. 278) and achieve a deeper understanding of life. The items of the reflective dimension mostly refer to questioning one's own role in difficulties, taking different perspectives on issues, and taking others' perspective in conflicts; two items concern emotion regulation in difficult situations. The *cognitive dimension* is defined as a person's competence to think deeply and learn about the difficult questions of human existence. All items of this dimension are reverse-coded to reduce social desirability. They refer to acceptance of simplified ideas and an unwillingness to think deeply about things. The *affective dimension* includes positive emotions and behaviors toward others and compassion with people in need (Ardelt, 2003). Three items refer to positive feelings for others, the others are about (reverse-coded) contempt or disinterest for other people, especially those in need of help.

The ASTI (Levenson et al., 2005) defines wisdom as self-transcendence, based upon Tornstam's construct of gerotranscendence (e.g., Tornstam, 1997) and Curnow's (1999) philosophical analysis of European and Asian wisdom literatures. Curnow identified four general principles of wisdom: self-knowledge, detachment, integration, and self-transcendence. Levenson et al. (2005) suggested to consider these principles as stages in the development of wisdom. Self-knowledge is awareness of the sources of one's sense of self. Detachment means to understand the transience and provisional nature of external sources such as relationships, roles, and material goods. Integration is the acceptance and inclusion of all self-aspects, including those that threaten self-illusions. Finally, self-transcendence means independence from external self-definitions and the dissolution of rigid boundaries between the self and others. It is difficult to assign the ASTI items unambiguously to these four components. They refer to inner peace independent of external things, feelings of unity with others and nature, joy in life, and an integrated sense of self.

The BWP is based on a definition of wisdom as expertise in the fundamental pragmatics of human life (e.g., Baltes and Staudinger, 2000). This expertise is measured by think-aloud tasks presenting participants with brief descriptions of a difficult life problem of a fictitious person. The problems refer to life review, life management, or life planning (Baltes and Staudinger, 2000). Response transcripts are evaluated by trained raters according to five criteria. *Factual knowledge* is knowledge about the nature and variability of human personality and experience, life-long development, and interpersonal relations. *Procedural knowledge* concerns strategies and heuristics for dealing with life problems. *Life-span contextualism* is the awareness and consideration of the influence of contexts and developmental stages on people's views and behavior. *Value relativism* refers to the awareness and acceptance of individual and cultural differences in value orientations. *Recognition and management of uncertainty* means an awareness of the inherent uncertainty and unpredictability of human life and the ability to deal with it constructively.

### Three types of wisdom measures

Our analysis of the content of researchers' definitions and operationalizations of wisdom suggests that the distinction between personal and general wisdom captures important differences between the measures. *Personal wisdom* pertains to what individuals have learned about themselves, others, and the world through their own experiences. This does not mean that personal wisdom is not applied to others, for example, when wise individuals mediate conflicts or give advice, but its basic source is introspection and (self-)reflection. The SAWS, the ASTI, and parts of the reflective component of the 3D-WS predominantly pertain to personal wisdom. *General wisdom* refers to wise ways of thinking about complex problems without an involvement of one's own self or particular concern for other people. The Berlin wisdom paradigm belongs into this category, as its tasks are about fictitious life problems of fictitious individuals and the rating criteria refer to how participants think about difficult issues in general. The cognitive dimension of the 3D-WS, which reflects a motivation to think deeply and beyond the obvious, also taps general wisdom.

There is one potentially important aspect of wisdom that may not be covered optimally by the distinction made above, namely, other-related wisdom. It refers to an empathy-based caring concern for both concrete other people and humankind at large. Other-related wisdom is the focus of the affective dimension of the 3D-WS. Aspects of other-related wisdom also come up in some items of the SAWS, the ASTI, and the reflective 3D-WS dimension that refer to taking others' perspective. Other-related wisdom is probably closer to personal than to general wisdom, as it refers to attitudinal and emotional aspects of personal relationships; in fact, Mickler and Staudinger (2008) suggested to measure personal wisdom by asking participants to think about themselves as a friend. Concern for others is a typical component of lay theories of wisdom (Clayton and Birren, 1980; Bluck and Glück, 2005), but laypeople do not unequivocally agree on its importance for wisdom (Glück and Bluck, 2011). In a Delphi survey, Jeste et al. (2010) found that wisdom researchers rated other-related aspects like altruism and generativity as highly characteristic of wisdom. In the definitions that wisdom researchers have proposed, however, other-related aspects are far less ubiquitous than cognitive or reflective aspects (Staudinger and Glück, 2011).

This classification suggests that the three types of measures should have different correlates. Measures of personal wisdom should be related to variables such as self-efficacy, openness to experience, personal growth, self-acceptance, and regulation of one's own emotions. Measures of general wisdom should be related to fluid and crystallized intelligence. Measures of other-related wisdom should be related to empathy and regulation of others' emotions. Previous evidence concerning correlates looks rather messy, however. For example, Taylor et al. (2011) found very similar correlate structures for the SAWS and the 3D-WS. The BWP is correlated to life experience, personal growth, affective engagement, and other-enhancing values (Staudinger et al., 1997, 1998; Kunzmann and Baltes, 2003). Thus, an alternative hypothesis is that all the measures tap a “wisdom syndrome” that binds self-, other-, and reasoning-related aspects together. In this vein, Ardelt (2003) argued that a self-reflective attitude leads people to develop wisdom-related knowledge as well as concern for others.

#### Face validity

In the context of content validity, it is important to also discuss questions of face validity. Whether a measure “looks like” it measures wisdom can have an important influence on the results: participants may want to present themselves, or actually view themselves, as wiser than they are. This is true for all self-report assessments of positive constructs, but it is particularly crucial with wisdom because wisdom includes self-reflection: truly wise persons are unlikely to declare themselves as wise (e.g., Assmann, 1994; Aldwin, 2009; Redzanowski and Glück, 2013), so they may score lower in self-report wisdom scales than others. Items referring to wisdom-related competencies, such as “I am good at identifying subtle emotions within myself” (SAWS), may be particularly susceptible to self-illusions, as most people are not very good at judging their own competencies (Freund and Kasten, 2012). Taylor et al. (2011) found a 0.26 correlation of the SAWS to positive self- deception.

The 3D-WS contains many reverse-coded items to reduce such self-presentation biases. However, disagreeing with a non-wisdom statement may not be the same as agreeing with a wisdom statement (for example, people might disagree with “Things often go wrong for me by no fault of my own” because they think things never go wrong for them), which could reduce internal consistency. While Ardelt (2003) found no relationship of the 3D-WS to social desirability, Taylor et al. (2011) found that the 3D-WS was correlated to both positive self-deception (*r* = 0.20) and impression management (*r* = 0.24). The reverse-coded items might also increase cognitive load, which could explain the negative correlation of the 3D-WS to age (Ardelt, 2003).

Correlations of the ASTI to social desirability are not available yet. It might be less susceptible to typical social desirability biases than the others because some of the items (e.g., “Whatever I do to others, I do to myself;” “I often have a sense of oneness with nature”) may simply not be accessible to individuals who have not reached a certain level of self-transcendence. This could be problematic, however, with relatively “unwise” samples who may find such items confusing.

The face-validity issues with self-report scales suggest the use of performance-based measures such as the BWP. Participants in BWP studies do not know the specific rating criteria or that they are being tested for wisdom. In fact, Glück and Baltes (2006) found that an instruction to give a wise response actually reduced performance in some participants. On the other hand, BWP participants may still produce what they think is a “good” response rather than what they actually think. Intelligent people may give a highly wise response to a fictitious life problem, but act much less wisely in a similar situation in their own life (Ardelt, 2004). Some authors have argued that performance measures of wisdom should focus on challenges in participants' own life (Ardelt, 2004; Glück et al., 2005; Glück and Bluck, 2013). Mickler and Staudinger's (2008) Bremen wisdom paradigm is an important step in this direction.

To summarize, the measures in this study conceptualize and operationalize wisdom in different ways, and the self-report measures differ in conceptual breadth and in the way they deal with self-presentation issues. In the following, we examine the reliability of the measures, their interrelations and factorial structure, and their relationships to relevant correlates.

### Reliability

Table 1 shows internal consistencies (Cronbach's alpha) for the self-report scales and inter-rater correlations for the BWP criteria. As expected from the scale-content analyses, internal consistencies, especially in relation to the number of items, were best for the SAWS (subscale Cronbach's alphas from 0.71 to 0.88; total-scale alpha: 0.90). They were also acceptable for the ASTI (0.83), the 3D-WS cognitive (0.74) and reflective (0.77) dimension, and the 3D-WS total score (0.86), suggesting that the larger number of items per subscale compensated for the broader range of item content in these measures. Internal consistency was least satisfactory for the 3D-WS affective dimension (alpha = 0.61). No single item accounted for this; exploratory factor analyses suggested that the subscale may contain at least three factors: two for the reverse-coded items (one referring to actual misanthropy, one to not caring about others' problems) and one for the three positive items. This finding suggests that disagreeing with a non-wise statement is not necessarily the unipolar opposite of agreeing to a wise statement.

The inter-rater correlations for the BWP were also largely acceptable, with the possible exceptions of life-span contextualism (r = 0.47) and recognition and management of uncertainty (0.53). They were lower than in other studies (e.g., Staudinger and Baltes, 1996; Glück and Baltes, 2006), which may suggest that students, even if carefully trained and calibrated, are less optimal raters for the BWP than middle-aged academics. Cronbach's alpha for the total BWP score (summed up across the ten ratings), however, was a satisfactory 0.85.

### Structural relationships

All correlations among the measures were significant, but only those of the ASTI with the SAWS (*r* = 0.50, *p* < 0.001) and the 3D-WS (*r* = 0.58, *p* < 0.001) were higher than 0.30. The 3D-WS had a correlation of only 0.26 (*p* = 0.001) to the SAWS. The BWP was correlated in this range to all three self-report measures (SAWS: *r* = 0.23, *p* = 0.024; ASTI: *r* = 0.30, *p* = 0.004; 3D-WS: *r* = 0.25, *p* = 0.018).

As Table 2 shows, the correlations between subscales were mostly higher within than across measures. In particular, the three 3D-WS dimensions were highly correlated. As its general-wisdom content would suggest, the cognitive dimension was also correlated to most BWP criteria. Interestingly, SAWS Openness was more highly correlated to the 3D-WS affective dimension (cf. Ardelt, 2011) and the ASTI than to the other SAWS subscales. The ASTI seemed to tap a broad range of aspects; it was correlated to 10 of the 13 subscales in the study. Correlations among the self-report subscales tended to be higher than between them and the BWP, but there were also a number of zero correlations, and even two significant negative correlations (both between SAWS Reminiscence and the 3D-WS). Thus, self-report as a method did not seem to explain much of the variance.

**Table 2:** **Correlations among wisdom subscales and BWP criteria**.

**Subscale/Criterion**	**2**	**3**	**4**	**5**	**6**	**7**	**8**	**9**	**10**	**11**	**12**	**13**	**14**
1 SAWS critical life experience	0.49^**^	0.52^**^	0.38^**^	0.26^**^	0.36^**^	−0.07	0.09	−0.04					
2 SAWS emotional regulation		0.78^**^	0.27^**^	0.48^**^	0.44	0.15^*^	0.17^*^	−0.03					
3 SAWS reminiscence & reflectiveness			0.15^*^	0.30^**^	0.13	−0.16^*^	−0.02	−0.22^**^	0.22^*^				
4 SAWS openness				0.36^**^	0.48^**^	0.38^**^	0.52^**^	0.39^**^					0.26^*^
5 SAWS humor					0.49^**^	0.24^**^	0.22^**^	0.14			0.26^*^		0.23^*^
6 ASTI						0.48^**^	0.45^**^	0.33^**^			0.25^*^	0.28^**^	0.40^**^
7 3D-WS reflective dimension							0.57^**^	0.58^**^				0.25^*^	0.33^**^
8 3D-WS affective dimension								0.44^**^					
9 3D-WS cognitive dimension										0.27^**^	0.23^*^	0.39^**^	0.49^**^
10 BWP uncertainty										0.41^**^	0.56^**^	0.52^**^	0.39^**^
11 BWP value relativism											0.57^**^	0.51^**^	0.47^**^
12 BWP life-span contextualism												0.76^**^	0.50^**^
13 BWP factual knowledge													0.69^**^
14 BWP procedural knowledge													

Only significant correlations are displayed. Correlations with the BWP are based only on the nominee and parallel control group (N = 94).

* p < 0.05,

** p < 0.01.

To test whether the classification into personal, general, and other-related types of wisdom accounted for the correlations, we used exploratory factor analyses with oblique rotation. (A confirmatory factor analysis did not reach satisfactory fit even when several cross-loadings across factors were permitted.) To determine the number of factors, we used several indicators that all converged on the three-factor solution: a scree plot, eigenvalues above 1, Velicer's MAP test, and parallel analysis (O'Connor, 2000). Together, the three factors explaining 64.6% of the variance. As Table 3 shows, the factors largely represented the SAWS, the 3D-WS, and the BWP, with the ASTI loading highest on the SAWS factor but cutting across all three. This structure partly supports the classification into personal, other-related, and general wisdom. The SAWS Openness scale loaded more strongly with the 3D-WS than with the SAWS. The BWP Procedural Knowledge criterion also had a second loading on the 3D-WS factor. As expected, the 3D-WS cognitive dimension had a second loading on the BWP factor. The correlations between the three factors were low (*r*_SAWS factor − 3D - WS factor_ = 0.11; *r*_SAWS factor − BWP factor_ = 13; *r*_3D - WS factor − BWP factor_ = 0.17). Thus, there is not much evidence for a strong common factor, but the common variance that there is seems to be captured well by the ASTI.

**Table 3:** **Loadings of the subscales and the BWP citeria in a factor analysis with oblique rotation**.

**Subscale/Criterion**	**Factor**		
	**1**	**2**	**3**
SAWS critical life experience	0.047	**0.702**	0.220
SAWS emotional regulation	0.165	**0.869**	−0.007
SAWS reminiscence and reflectiveness	0.119	**0.814**	−0.230
SAWS openness	0.115	**0.410**	**0.682**
SAWS humor	0.174	**0.589**	0.277
ASTI	**0.303**	**0.580**	**0.565**
3D-WS reflective dimension	0.218	−0.022	**0.782**
3D-WS affective dimension	−0.016	0.202	**0.833**
3D-WS cognitive dimension	**0.372**	−0.205	**0.753**
BWP uncertainty	**0.711**	0.209	−0.123
BWP value relativism	**0.758**	0.052	0.127
BWP contextualism	**0.850**	0.149	0.104
BWP factual knowledge	**0.865**	0.093	0.268
BWP procedural knowledge	**0.743**	0.133	**0.408**

Analysis included only the nominee group and parallel control group (N = 94). Loadings above 0.30 are in bold print.

### Construct validity

To assess construct validity, we related the four wisdom measures to (a) three alternative indicators of wisdom: nomination for wisdom and two interview-based wisdom ratings, (b) self-related, other-related, and cognitive correlates of wisdom, and (c) age. We also analyzed relationships to gender and education.

#### Relationships to alternative indicators of wisdom

We first compared the wisdom nominees in the sample to the control group. It is important to note that nomination has limited validity as an indicator of wisdom because there were large differences in the actual familiarity of the nominators with the nominees. Nevertheless, wisdom nominees scored higher than control participants in all four measures, as Table 4 shows. When education and vocabulary were controlled, the group differences remained virtually unchanged for the self-report measures, but became only marginally significant for the BWP.

**Table 4:** **Differences between wisdom nominees and control participants in the four wisdom measures**.

**Wisdom measure**	**M (*SD*) nominees**	**M (*SD*) controls**	***T*-test**	**Controlling for education and vocabulary**
**SAWS**	4.62 (0.50)	4.27 (0.51)	*t* (167) = 3.95 *R*^2^ = 0.085, *p* < 0.001	*R*^2^ = 0.082, *p* = 0.001
**ASTI**	3.35 (0.33)	3.07 (0.30)	*t* (162) = 5.14 *R*^2^ = 0.140, *p* < 0.001	*R*^2^ = 0.135, *p* < 0.001
**3D-WS**	3.82 (0.41)	3.60 (0.39)	*t* (167) = 3.34 *R*^2^ = 0.063, *p* = 0.001	*R*^2^ = 0.059, *p* = 0.004
**BWP**	3.17 (1.07)	2.62 (1.02)	*t* (91) = 2.50 *R*^2^ = 0.064, *p* = 0.014	*R*^2^ = 0.046, *p* = 0.057

Concerning the wisdom ratings of the interviews, we expected the rating for the difficult-event interview to be more strongly correlated to measures of personal wisdom, because the interview questions concerned the participant's own view, and the conflict-narrative rating to measures of other-related wisdom, because this interview included the opponent's perspective. Confirming these expectations, the SAWS and the ASTI were significantly correlated to the difficult-event interview rating (SAWS: *r* = 0.26, *p* = 0.012, ASTI: *r* = 0.32, *p* = 0.003), whereas the 3D-WS was significantly correlated to the conflict interview rating (*r* = 0.26, *p* = 0.014). The BWP score was significantly correlated to the conflict-interview rating (*r* = 0.22, *p* = 0.044) and marginally to the difficult-event interview (*r* = 0.20, *p* = 0.051), which may be partly due to common method variance among interview-based measures (note that the BWP ratings and the interview-transcript ratings were provided by different raters).

#### Relationships to correlates of wisdom

Table 5 shows the correlations between the wisdom measures and the self-related, other-related, and cognitive correlates.

**Table 5:** **Correlations between wisdom measures and correlates**.

**Correlate**	**SAWS**	**3D-WS**	**ASTI**	**BWP**
**SELF-RELATED**				
Self-Efficacy	0.384^**^	0.329^**^	0.335^**^	0.113
Openness to experience	0.409^**^	0.591^**^	0.444^**^	0.365^**^
Ryff personal growth	0.282^**^	0.413^**^	0.222^**^	0.173
Ryff self-acceptance	0.170^*^	0.369^**^	0.327^**^	0.003
Emotional competence/self	0.317^**^	0.627^**^	0.500^**^	0.276^**^
**OTHER-RELATED**				
Empathy	0.394^**^	0.260^**^	0.282^**^	−0.012
Emotional competence/others	0.448^**^	0.482^**^	0.467^**^	0.265^*^
**COGNITIVE**				
Inductive reasoning	−0.154^*^	0.223^**^	−0.017	0.104
Vocabulary	0.176^*^	0.135	0.116	0.126

Correlations with the BWP are based only on the nominee and parallel control group (N = 94).

* p < 0.05,

** p < 0.01.

The correlations did not support the distinction of personal and other-related self-report measures. For example, the highest correlation for the SAWS was with emotional competence concerning others, which was also highly correlated to the ASTI and the 3D-WS. There were hardly any clear differences between the three scale measures; if anything, the SAWS seemed to be more highly correlated to empathy and less highly to self-acceptance than the two others. Generally, the correlations tended to be higher for the 3D-WS than for the other two self-report measures. The BWP had significant correlations to openness to experience and the emotional-competence measures, but to neither of the two intelligence measures.

In order to get a clearer picture, we performed an exploratory factor-analysis of the self-report correlates with oblique rotation. Here, the scree plot, eigenvalue criterion, and parallel analysis suggested two factors whereas the MAP test suggested only one factor. To obtain more differentiated results, we used two factors, which explained 58.8% of the variance. The two factors largely represented the self-related/other-related distinction, with self-efficacy (loading = 0.73), self-acceptance (0.77), and self-related emotion regulation (0.80) loading on the first factor, empathy (0.76) and openness to experience (0.77) loading on the second factor, and personal growth (0.52, 0.55) and other-related emotion regulation (0.52, 0.62) loading on both factors. Both factor scores were significant predictors of the SAWS (*r*^2^ = 0.33; β_self_ = 0.26, *p* < 0.001, β_other_ = 0.46, *p* < 0.001), the 3D-WS (*r*^2^ = 0.51; β_self_ = 0.48, *p* < 0.001, β_other_ = 0.44, *p* < 0.001), and the ASTI (*r*^2^ = 0.34; β_self_ = 0.39, *p* < 0.001, β_other_ = 0.35, *p* < 0.001). They did not predict the BWP, although there was a marginal relation for the other-related factor (*r*^2^ = 0.09; β_self_ = 0.14, *p* = 0.225, β_other_ = 0.22, *p* = 0.054). Again, the amount of common variance was somewhat higher for the 3D-WS than for the SAWS and the ASTI, and the SAWS was more strongly related to the other-related than to the self-related correlates in spite of its largely self-related content.

Correlations to intelligence were low, and insignificant for the ASTI and the BWP. The SAWS was positively related to vocabulary (*r* = 0.18, *p* = 0.024) and negatively to inductive reasoning (*r* = −0.15, *p* = 0.050); both became insignificant when age was controlled. In contrast, the 3D-WS was positively correlated to inductive reasoning (*r* = 0.22, *p* = 0.004), and this relationship remained significant after controlling for age. Specifically, the reflective (*r* = 0.29, *p* < 0.001) and the cognitive (*r* = 0.31, *p* < 0.001) but not the affective dimension were correlated to inductive reasoning.

#### Relationships to age, gender, and education

Finally, relationships to age, gender, and education were analyzed. While wisdom does not generally increase with age, both laypeople (Glück and Bluck, 2011) and researchers would expect most highly wise people to be at least in their sixties (Staudinger, 1999; Glück and Bluck, 2013). Thus, a valid measure of wisdom need not be positively correlated to age, but it should not be negatively correlated either, and the highest-scoring individuals should be older than the others.

**Figure 2:**
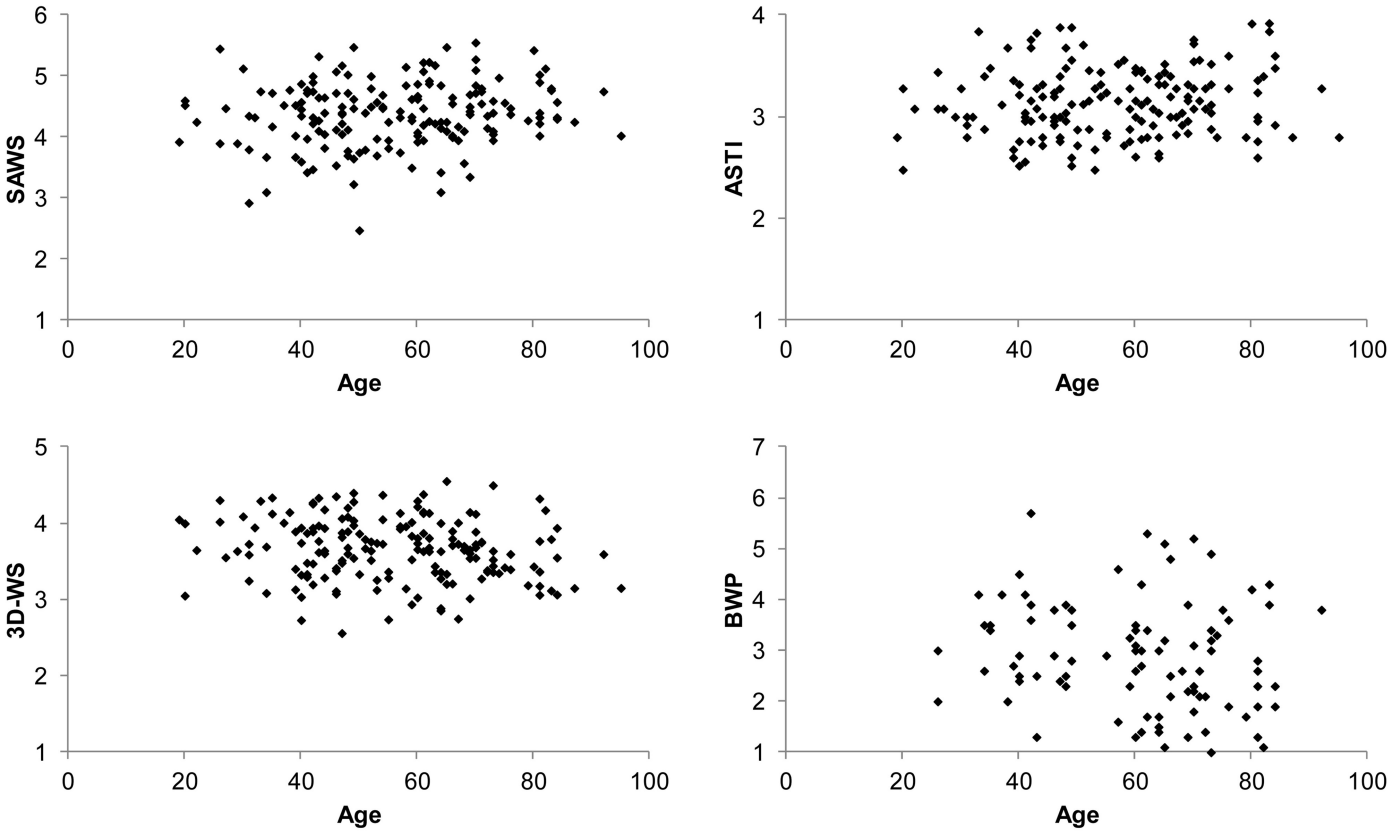
**Relationship between the wisdom measures and age**.

Figure 2 shows scatterplots of the four wisdom measures with age. The y axis displays the full range of possible scores (means across the scale items) for each measure. As the figure shows, the self-report scores were largely in the upper half of the respective scales, whereas BWP scores were largely in the lower half. Correlations to age were insignificant for the ASTI (*r* = 0.12, *p* = 0.144) and the BWP (*r* = −0.16, *p* = 0.132), positive and marginally significant for the SAWS (*r* = 0.15, *p* = 0.052), and negative and significant for the 3D-WS (*r* = −0.17, *p* = 0.025). Specifically, there was a correlation of −0.39 (*p* < 0.001) for the cognitive dimension of the 3D-WS and insignificant correlations for the affective and reflective dimension. In none of the four measures were the top 25% scorers older than the other participants; in the 3D-WS they were significantly younger, *t*(167) = 2.54, *p* = 0.012.

Concerning gender, current wisdom theories do not point to any general differences between men and women, and there were no significant differences in any of the measures. Education can be viewed as a meaningful correlate of wisdom if the development of wisdom entails a strong learning motivation (Ardelt, 2003). At the same time, especially with highly “verbal” measures, it may also be an unwanted confound. In the current study, participants at the three educational levels in the Austrian school system (compulsory 9 years; high-school; college or university) did not differ in the ASTI and SAWS. Participants with higher education scored significantly higher in the 3D-WS, *F*_(2, 140)_ = 3.10, *p* = 0.048, and the BWP, *F*_(2, 83)_ = 3.84, *p* = 0.034. For the 3D-WS, the relationship to education was again strongest for the cognitive dimension, *F*_(2, 140)_ = 4.70, *p* = 0.011. Concerning the BWP, education was significantly related to procedural knowledge, *F*_(2, 83)_ = 5.72, *p* = 0.011, and value relativism, *F*_(2, 83)_ = 6.66, *p* = 0.001.

## A brief wisdom screening scale

Finally, we used a purely empirical approach to develop a “brief wisdom screening scale” (BWSS) by identifying those 20 items from the three self-report scales that had the highest correlations to the common factor across the scales. For cross-validation, we randomly divided the sample into two subsamples (N_1_ = 88, N_2_ = 82). In the first subsample, we factor-analyzed the total scores of the SAWS, ASTI, and 3D-WS, resulting in a strong general “wisdom self-report” factor that explained 82.9% of the variance, and computed a factor score. Of the 114 items, 90 had significant correlations to the factor score; Table 6 lists the 20 items with the highest correlations. As the table shows, the items come from all three scales and most subscales, and cover a broad range of facets of wisdom.

**Table 6:** **The brief wisdom screening scale: 20 items with the highest correlations (*r*) to the general “self-reported wisdom” factor**.

**Item**	***r***	**Item wording**	**Aspect of wisdom**
ASTI 30	0.60	I am able to integrate the different aspects of my life.	Self-transcendence
SAWS 37	0.59	It seems I have a talent for reading other people's emotions.	Emotional regulation
ASTI 13	0.59	I have a good sense of humor about myself.	Self-transcendence
SAWS 22	0.56	I can freely express my emotions without feeling like I might lose control.	Emotional regulation
ASTI 32	0.54	I can accept the impermanence of things.	Self-transcendence
3D-WS 14b	0.53	Sometimes I get so charged up emotionally that I am unable to consider all ways of dealing with my problems. (reversed)	Reflective dimension
ASTI 33	0.52	I have grown as a result of losses I have suffered.	Self-transcendence
SAWS 35	0.52	I'm very curious about other religious and/or philosophical belief systems.	Openness
SAWS 24	0.51	At this point in my life, I find it easy to laugh at my mistakes.	Humor
ASTI 7	0.50	My peace of mind is not easily upset.	Self-transcendence
ASTI 11	0.50	My happiness is not dependent on other people and things.	Self-transcendence
SAWS 36	0.50	I've learned valuable life lessons from others.	Reminiscence and reflectiveness
ASTI 3	0.49	I don't worry about other people's opinions of me.	Self-transcendence
3D-WS 11b	0.49	I either get very angry or depressed if things go wrong. (reversed)	Reflective dimension
SAWS 5	0.48	I like to read books which challenge me to think differently about issues.	Openness
ASTI 2	0.48	I feel that my individual life is a part of a greater whole.	Self-transcendence
3D-WS 5b	0.48	I always try to look at all sides of a problem.	Reflective dimension
ASTI 22	0.47	I often have a sense of oneness with nature.	Self-transcendence
SAWS 12	0.46	I am “tuned in” to my own emotions.	Emotional regulation
3D-WS 8a	0.45	There are some people I know I would never like. (reversed)	Affective dimension
SAWS 11	0.45	I have dealt with a great many different kinds of people during my lifetime.	Critical life experience

As the original items had different response scales, all item scores were transformed to values between 0 and 1, and the mean was used as BWSS score. Expectably, the correlation between the BWSS score and the wisdom general-factor score was very high (*r* = 0.90) in Subsample 1; the crucial question now was whether the item selection would cross-validate in Subsample 2. For this purpose, an independent factor analysis was performed in Subsample 2, identifying a general factor that explained 65.0% of the variance. The correlation of the BWSS sum score to the new factor score in Subsample 2 was 0.92 (*r* < 0.001). Thus, the Brief Wisdom Screening Scale was representative of the general wisdom factor in both subsamples.

Cronbach's alpha for the BWSS was 0.87 in both subsamples, as well as in the total sample. The BWSS was, expectably, highly correlated to the scores in the three original self-report scales (SAWS: *r* = 0.60, ASTI: *r* = 0.81, 3D-WS: *r* = 0.75; all *p*s < 0.001), but it was also significantly correlated to the BWP score (*r* = 0.21, *p* = 0.047), wisdom nomination (*r* = 0.39, *p* < 0.001), and the mean wisdom ratings for the conflict interview (*r* = 0.26, *p* = 0.014) and the difficult-event interview (*r* = 0.21, *p* = 0.045). The correlations to the correlates of wisdom were all significant (self-efficacy: *r* = 0.44, *p* < 0.001; openness to experience: *r* = 0.50, *p* < 0.001; personal growth: *r* = 0.32, *p* < 0.001; self-acceptance: *r* = 0.47, *p* < 0.001; emotional competence/self: *r* = 0.63, *p* < 0.001; empathy: *r* = 0.28, *p* < 0.001; emotional competence/others: *r* = 0.54, *p* < 0.001; vocabulary: *r* = 0.20, *p* = 0.011), except for inductive reasoning (*r* = 0.04, *p* = 0.575). The BWSS was not significantly related to gender, *r* = 0.004 (*p* = 0.963) or age (*r* = 0.10, *p* = 0.204), but marginally positively to education, *r* = 0.16 (*p* = 0.057).

## Discussion

This study analyzed the content, reliability, structural relationships, and validity of four well-established measures: the SAWS (Webster, 2003), the 3D-WS (Ardelt, 2003), the ASTI (Levenson et al., 2005), and the Berlin wisdom paradigm (overview in Baltes and Staudinger, 2000). Reliability, structural relationships, and validity of the measures were investigated in a sample of 47 wisdom nominees and 123 control participants. Based on a content analysis, three different “types” of wisdom were identified: personal wisdom, general wisdom, and other-related wisdom. Reliability was highest for the SAWS subscales and at the lower limit of acceptability for the affective dimension of the 3D-WS and two BWP criteria. Correlations between the measures were only in the 0.20 s with the exception of the ASTI, which had correlations above 0.50 to the two other self-report measures and significant correlations to most subscales and criteria of the other measures. A factor analysis of the subscales identified three factors largely representing the SAWS, the 3D-WS, and the BWP, with the ASTI cutting across all three.

Concerning construct validity, all measures were significantly related to wisdom nomination and interview-based wisdom ratings. All three self-report measures were significantly correlated to all self-report correlates of wisdom. While this broad range of correlations could be expected for the 3D-WS, which taps all three types of wisdom (and for which the amount of variance explained by the correlates was highest), it came somewhat unexpected for the SAWS, which had higher correlations to other-related than to self-related correlates although its content identified it as a measure of personal wisdom. Thus, even those measures that focus on personal wisdom seem to tap a “wisdom syndrome” that includes other-related aspects. As Ardelt (2003) argued, a self-reflective attitude may be underlying the development of cognitive and affective aspects of wisdom including self-transcendence.

The only performance measure in this study, the Berlin wisdom paradigm, was correlated only to openness to experience and emotional competence among the self-report correlates; unexpectedly and in contrast to other studies (Staudinger et al., 1998), it was also uncorrelated to measures of intelligence. These differences may to some degree be due to the limited reliability of the BWP in this study.

Some cautionary remarks about the current sample are necessary. To increase the likelihood of finding high levels of wisdom, we included a group of wisdom nominees. However, the nature of the main measures of the study—interviews about highly difficult autobiographical experiences—probably also led to a relatively wise control group: the fact that only about 10% of the participants who were invited for the control group agreed to participate suggests that those who did participate were probably more interested in difficult life matters than the general population is. Therefore, the variances of the wisdom measures may be somewhat underestimated. Our impression from the interviews, however, was that few of the participants in either group displayed impressive levels of wisdom.

### Recommendations for researchers

Researchers looking for a measure of wisdom should first decide which type(s) of wisdom they find most central for their study: personal wisdom, general wisdom, or other-related wisdom. A second question is whether they consider self-report a valid method for measuring wisdom or if they want to use a performance measure, which requires more time and effort.

#### Measures of personal wisdom

The SAWS (Webster, 2007) measures personal wisdom in five subscales: Critical Life Experience, Emotional Regulation, Reminiscence and Reflectiveness, Openness, and Humor (the latter is not tapped by any of the other available measures of wisdom, nor by most of the theoretical wisdom literature, which we consider a flaw of the wisdom literature rather than the SAWS). The subscales are highly reliable but may be somewhat narrow in scope (see also Ardelt, 2011); Openness taps other-related as well as personal wisdom. The SAWS score was positively related to self-related as well as other-related correlates of wisdom. It had a low positive correlation to age, a low negative correlation to inductive reasoning, and a low positive correlation to vocabulary; the latter two became insignificant when age was controlled for. Thus, the SAWS may tap typical processes of lifespan cognitive development, with losses in fluid intelligence and stability or growth in crystallized intelligence over the life span (e.g., Baltes et al., 1999). A potentially critical aspect concerns the high face validity of the SAWS, especially those items that assess competencies by self-report. Therefore, the SAWS might be combined with a social desirability scale.

The ASTI (Levenson et al., 2005) may seem, at first glance, conceptually narrower than the two other self-report scales because it defines wisdom as only one thing—self-transcendence. The items of the ASTI, however, refer to self-knowledge, detachment, self-integration, and self-transcendence. This is probably the reason why the internal consistency of the ASTI is acceptable but not extremely high. The ASTI score seems to tap the “essence” of wisdom across all measures particularly well, as it was correlated to ten of the 13 wisdom subscales in this study and all personality and affect correlates. The ASTI was unrelated to intelligence, age, or education. It presents itself as a good option for researchers who theoretically accept the idea of self-transcendence as a core aspect of wisdom—a conceptual approach that has been somewhat outside the “mainstream” of wisdom research although it is based on Curnow's (1999) philosophical analysis of wisdom across cultures. Our findings support the idea that self-transcendence is central to wisdom. The ASTI may be somewhat problematic, however, with relatively “wisdom-distant” participants who may have difficulty understanding some of the items.

#### A measure including other-related wisdom

The 3D-WS (Ardelt, 2003) spans a particularly broad range of aspects of wisdom, as it includes a reflective (self-related), a cognitive (general), and an affective (other-related) dimension; as a whole, it has a stronger focus on other-related aspects than the other measures. The internal consistency of the affective dimension was limited at least in the German version used here (see also Redzanowski and Glück, 2013). The 3D-WS was correlated to SAWS Openness, the ASTI, and, concerning the cognitive dimension, subscales of the BWP. It had the highest correlations to correlates of wisdom; in fact, it was more highly correlated to self-related emotion regulation and openness to experience than to the other wisdom measures. Interestingly, however, the highest correlations were with self-related, rather than other-related, correlates, which may partly be due to the low internal consistency of the affective (other-related) dimension. A potentially problematic aspect is the large number of reverse-coded items, which may reduce reliability and produce a significant influence of fluid intelligence. Supporting this notion, both the reflective and the cognitive dimension were correlated to inductive reasoning, and the cognitive dimension was negatively related to age and positively to education. Thus, researchers might want to combine the 3D-WS with a brief measure of fluid intelligence.

#### A performance measure of general wisdom

The BWP (Baltes and Smith, 1990), the only performance measure in this study, requires much more time, effort, and expenses than the self-report measures: participants need to be interviewed by trained interviewers, including practice tasks, response protocols need to be recorded and transcribed, and transcripts need to be evaluated by ten independent trained raters. In previous studies, we have tried to reduce costs by using written versions or reducing the number of raters, but neither worked well. In the current work, raters were students, rather than middle-aged academics, which may have affected reliability and validity. Apart from questions of cost and effort, the BWP largely taps general wisdom, that is, participants' ability to think wisely about problems unrelated to their own life. Researchers looking for a performance measure of personal wisdom should consider Mickler and Staudinger's (2008) Bremen Wisdom Paradigm. In spite of this focus, scores in the BWP are also correlated to self- and other-related variables (Staudinger et al., 1997, 1998; Kunzmann and Baltes, 2003), though not to the same degree as the self-report measures. In the current study, the relationships to (self-report) measures of those aspects were much lower than for the self-report wisdom measures, with the exception of openness to experience. BWP performance was also related to education, which may be typical for language-based measures, as well as to interview-based wisdom ratings and wisdom nomination.

The *Brief Wisdom Screening Scale* introduced here is a purely empirical compilation of those 20 items from the three self-report scales that had the highest correlations to the general factor. It spans a broad range of content, from interest in philosophy to feeling unity with nature or being tuned in to one's own emotions; thus, it does not represent any particular theory of wisdom or allow for an analysis of facets of wisdom. Although the item selection was cross-validated in an independent subsample, more research is certainly needed to establish the usefulness of the scale in other populations. For the time being, we suggest to use it only as a screening measure of wisdom in studies that focus on other variables. We recommend to present the BWSS items in a random order and with a five-point Likert response scale from “strongly disagree” to “strongly agree.”

### The current state of wisdom research

In sum, although the measures of wisdom studied here differ markedly in theoretical background and content, their relationships to other variables did not differ that much. For other studies, however, it may still be important to select the wisdom measure that makes the best conceptual and empirical sense. While all measures used in this study (except the BWSS) are based on convincing theoretical foundations and have been carefully developed and tested by their authors, none is entirely convincing as a measure of wisdom. For example, the relationship to age that developmental theories of wisdom would suggest—the wisest individuals should be somewhat older than the rest—was not found for any of the measures. While the idea of the “wise old person” may be a stereotype rather than an empirical truth, we tend to believe that the problem is in the nature of the measurement approaches. The strengths of (some) older people may manifest when they display wisdom in real life rather than in wisdom scales or tasks, because neither produce any serious emotional involvement. While it is obviously unethical to put people into emotionally challenging situations to measure their wisdom, approaches that utilize autobiographical accounts of real-life experiences may be a promising new avenue (Ardelt, 2004; Glück et al. submitted).

It is important to note that several existing measures of wisdom were not included in this study. For example, Brown and Greene (2006; Greene and Brown, 2009) have published an interesting self-report scale based on a developmental model of wisdom. Mickler and Staudinger's (2008) Bremen Wisdom Paradigm is a highly promising performance measure of personal wisdom. New experimental work also suggests avenues for the development of wisdom measures; for example, Grossmann et al. (2010) found positive relations to age for a social reasoning task in which participants predicted how interpersonal conflicts would unfold. Still, the number of wisdom measures available is not too impressive yet. In particular, we need measures of other-related wisdom and non-self-report measures of wisdom that do not require extensive coding effort. We hope that this work may encourage creative researchers to join our quest for new ways to measure wisdom.

### Conflict of interest statement

The authors declare that the research was conducted in the absence of any commercial or financial relationships that could be construed as a potential conflict of interest.

## References

[B1] Aldwin C. (2009). Gender and wisdom: a brief overview. Res. Hum. Dev. 6, 1–8 10.1080/15427600902779347

[B2] Ardelt M. (2000). Intellectual versus wisdom-related knowledge: the case for a different kind of learning in the later years of life. Educ. Gerontol. 26, 771–789 10.1080/036012700300001421

[B3] Ardelt M. (2003). Empirical assessment of a three-dimensional wisdom scale. Res. Aging 25, 275–324 10.1177/0164027503025003004

[B4] Ardelt M. (2004). Wisdom as expert knowledge system: a critical review of a contemporary operationalization of an ancient concept. Hum. Dev. 47, 257–285 10.1159/000079154

[B5] Ardelt M. (2011). The measurement of wisdom: a commentary on Taylor, Bates, and Webster's comparison of the SAWS and 3D-WS. Exp. Aging Res. 37, 241–255 10.1080/0361073X.2011.554509 21424959

[B6] Assmann A. (1994). “Wholesome knowledge: concepts of wisdom in a historical and cross-cultural perspective,” in Life-span Development and Behavior, Vol. 12, eds Featherman D. L. Lerner R. M. Perlmutter M. (Hillsdale, NJ: Erlbaum), 187–224

[B7] Baltes P. B. Smith J. (1990). “Toward a psychology of wisdom and its ontogenesis,” in Wisdom: Its Nature, Origins, and Development, ed Sternberg R. J. (New York, NY: Cambridge University Press), 87–120 10.1017/CBO9781139173704.006

[B8] Baltes P. B. Staudinger U. M. (2000). Wisdom: a metaheuristic (pragmatic) to orchestrate mind and virtue towards excellence. Am. Psychol. 55, 122–136 10.1037/0003-066X.55.1.122 11392856

[B9] Baltes P. B. Staudinger U. M. Lindenberger U. (1999). Life-span developmental psychology. Annu. Rev. Psychol. 50, 471–507 10.1146/annurev.psych.50.1.471 15012462

[B10] Bluck S. Glück J. (2005). “From the inside out: people's implicit theories of wisdom,” in A Handbook of Wisdom: Psychological Perspectives, eds Sternberg R. J. Jordan J. (Cambridge: Cambridge University Press), 84–109 10.1017/CBO9780511610486.005

[B11] Borkenau P. Ostendorf F. (1993). NEO-Fünf-Faktoren-Inventar (NEO-FFI) nach Costa und McCrae. [NEO Five Factors Inventory (NEO-FFI) after Costa and McCrae]. Göttingen: Hogrefe

[B12] Brown S. C. Greene J. A. (2006). The wisdom development scale: translating the conceptual to the concrete. J. Coll. Stud. Dev. 47, 1–19 10.1353/csd.2006.0002

[B13] Clayton V. P. Birren J. E. (1980). “The development of wisdom across the lifespan: a reexamination of an ancient topic,” in Life-span Development and Behavior, Vol. 3, eds Baltes P. B. Brim O. G. (San Diego, CA: Academic Press), 103–135

[B14] Curnow T. (1999). Wisdom, Intuition, and Ethics. Aldershot: Ashgate

[B15] Davis M. H. (1983). Measuring individual differences in empathy: evidence for a multidimensional approach. J. Pers. Soc. Psychol. 44, 113–126 10.1037/0022-3514.44.1.113

[B16] Freudenthaler H. H. Neubauer A. C. (2005). Emotional intelligence: the convergent and discriminant validities of intra- and interpersonal emotional abilities. Pers. Individ. Diff. 39, 569–579 10.1016/j.paid.2005.02.004

[B17] Freudenthaler H. H. Neubauer A. C. Gabler P. Scherl W. G. Rindermann H. (2008). Testing and validating the trait emotional intelligence questionnaire (TEIQue) in a German-speaking sample. Pers. Individ. Diff. 45, 673–678 10.1016/j.paid.2008.07.014

[B18] Freund P. A. Kasten N. (2012). How smart do you think you are. A meta-analysis on the validity of self-estimated cognitive ability. Psychol. Bull. 138, 296–321 10.1037/a0026556 22181852

[B19a] Germer C. K. Siegel R. D. (Eds.) (2012). Wisdom and compassion in Psychotherapy: Deepening mindfulness in clinical practice. New York, NY: Guilford Press

[B19] Glück J. Baltes P. B. (2006). Using the concept of wisdom to enhance the expression of wisdom knowledge: not the philosopher's dream, but differential effects of developmental preparedness. Psychol. Aging 21, 679–690 10.1037/0882-7974.21.4.679 17201489

[B20] Glück J. Bluck S. (2007). Looking back across the lifespan: a life story account of the reminiscence bump. Mem. Cogn. 35, 1928–1939 10.3758/BF03192926 18265609

[B21] Glück J. Bluck S. (2011). Laypeople's conceptions of wisdom and its development: cognitive and integrative views. J. Gerontol. B Psychol. Sci. 66B, 321–324 10.1093/geronb/gbr011 21398417

[B22] Glück J. Bluck S. (2013). “MORE wisdom: a developmental theory of personal wisdom,” in The Scientific Study of Personal Wisdom eds Ferrari M. Weststrate N. (New York, NY: Springer), 75–98 10.1007/978-90-481-9231-1_4

[B23] Glück J. Bluck S. Baron J. McAdams D. (2005). The wisdom of experience: autobiographical narratives across adulthood. Int. J. Beh. Dev. 29, 197–208 10.1177/0165025044400050415102038

[B25] Greene J. A. Brown S. C. (2009). The wisdom development scale: further validity investigations. Int. J. Aging Hum. Dev. 68, 289–320 10.2190/AG.68.4.b 19711618

[B26] Grossmann I. Karasawa M. Izumi S. Na J. Varnum M. E. W. Kitayama S. (2013). Aging and wisdom: culture matters. Psychol. Sci. 23, 1059–1066 22933459 10.1177/0956797612446025

[B27] Grossmann I. Na J. Varnuma M. E. W. Park D. C. Kitayama S. Nisbett R. E. (2010). Reasoning about social conflicts improves into old age. Proc. Natl. Acad. Sci. U.S.A. 107, 7246–7250 10.1073/pnas.1001715107 20368436 PMC2867718

[B29a] Jeste D. V. Ardelt M. Blazer D. Kraemer H. C. Vaillant G. Meeks T. W. (2010). Expert consensus on characteristics of wisdom: a Delphi method study. Gerontologist 50, 668–680 10.1093/geront/gnq022 20233730 PMC2937249

[B29b] Kilburg R. R. (2012). Virtuous leaders: Strategy, character, and influence in the 21st century. Washington, DC: American Psychological Association

[B29] Kross E. Grossmann I. (2012). Boosting wisdom: distance from the self enhances wise reasoning, attitudes, and behavior. J. Exp. Psychol. 141, 43–48 10.1037/a0024158 21728454

[B30] Kunzmann U. Baltes P. (2003). Wisdom-related knowledge: affective, motivational, and interpersonal correlates. Pers. Soc. Psychol. Bull. 29, 1104–1119 10.1177/0146167203254506 15189607

[B31] Le T. Levenson M. R. (2005). Wisdom: what's love (and culture) got to do with it. J. Res. Pers. 39, 443–457 10.1016/j.jrp.2004.05.003

[B32] Lehrl S. (2005). Mehrfach-Wortschatz Intelligenz Test MWT-B. [Multiple-choice Vocabulary Intelligence Test MWT-B]. Balingen: Spitta Verlag

[B33] Levenson R. Jennings P. A. Aldwin C. Shiraishi R. W. (2005). Self-transcendence, conceptualization and measurement. Int. J. Aging Hum. Dev. 60, 127–143 10.2190/XRXM-FYRA-7U0X-GRC0 15801386

[B34] Mickler C. Staudinger U. M. (2008). Personal wisdom: validation and age-related differences of a performance measure. Psychol. Aging 23, 787–799 10.1037/a0013928 19140650

[B35] O'Connor B. (2000). SPSS and SAS programs for determining the number of components using parallel analysis and Velicer's MAP test. Beh. Res. Methods Instrum. Comput. 32, 396–402 11029811 10.3758/bf03200807

[B36] Redzanowski U. Glück J. (2013). Who knows who is wise. Self- and peer-ratings of wisdom. J. Gerontol. B Psychol. Sci. 68, 391–394 10.1093/geronb/gbs079 22967506

[B37] Ryff C. D. Keyes C. L. M. (1995). The structure of psychological well-being revisited. J. Pers. Soc. Psychol. 69, 719–727 10.1037/0022-3514.69.4.719 7473027

[B38] Schwarzer R. Jerusalem M. (1995). “Generalized Self-Efficacy scale,” in Measures in Health Psychology: A User's Portfolio. Causal and Control Beliefs, eds Weinman J. Wright S. Johnston M. (Windsor: NFER-NELSON), 35–37

[B39] Staudinger U. M. (1999). Older and wiser. Integrating results on the relationship between age and wisdom-related performance. Int. J. Behav. Dev. 23, 641–664 10.1080/016502599383739

[B40] Staudinger U. M. Baltes P. B. (1996). Interactive minds: a facilitative setting for wisdom-related performance. J. Pers. Soc. Psychol. 71, 746–762 10.1037/0022-3514.71.4.746

[B41] Staudinger U. M. Dörner J. Mickler C. (2005). “Wisdom and personality,” in A Handbook of Wisdom: Psychological Perspectives, eds Sternberg R. J. Jordan J. (New York: Cambridge University Press), 191–219 10.1017/CBO9780511610486.009

[B42] Staudinger U. M. Glück J. (2011). Psychological wisdom research: commonalities and differences in a growing field. Annu. Rev. Psychol. 62, 215–241 10.1146/annurev.psych.121208.131659 20822439

[B43] Staudinger U. M. Lopez D. Baltes P. B. (1997). The psychometric location of wisdom-related performance: intelligence, personality, and more. Pers. Soc. Psychol. Bull. 23, 1200–1214 10.1177/01461672972311007

[B44] Staudinger U. M. Maciel A. G. Smith J. Baltes P. B. (1998). What predicts wisdom-related performance. A first look at personality, intelligence, and facilitative experiential contexts. Eur. J. Pers. 12, 1–17

[B45] Staudinger U. M. Smith J. Baltes P. B. (1994). Manual for the Assessment of Wisdom-related Knowledge. Berlin: Max Planck Institute for Human Development

[B46a] Sternberg R. J. (2010). WICS: a new model for school psychology. Sch. Psychol. Int. 31, 599–616 10.1177/0143034310386534

[B46] Taylor M. Bates G. Webster J. D. (2011). Comparing the psychometric properties of two measures of wisdom: predicting forgiveness and psychological well-being with the self-assessed wisdom scale (SAWS) and the three-dimensional wisdom scale (3D-WS). Exp. Aging Res. 37, 129–141 10.1080/0361073X.2011.554508 21424954

[B47] Tornstam L. (1997). Gerotranscendence – the contemplative dimension of aging. J. Aging Stud. 11, 143–154 10.1016/S0890-406590018-9

[B48] Webster J. D. (2003). An exploratory analysis of a self-assessed wisdom scale. J. Adult Dev. 10, 13–22 10.1023/A:102078261905117957986

[B49] Webster J. D. (2007). Measuring the character strength of wisdom. Int. J. Aging Hum. Dev. 65, 163–183 10.2190/AG.65.2.d 17957986

[B50] Webster J. D. (2010). Wisdom and positive psychosocial values in young adulthood. J. Adult Dev. 17, 70–80 10.1007/s10804-009-9081-z

[B51] Weiss R. H. (2008). Grundintelligenztest Skala 2 - Revision - (CFT 20-R) mit Wortschatztest und Zahlenfolgentest. [Basic Intelligence Test Scale 2 – Revised (CFT 20-R) with Vocabulary and Counting Test]. Göttingen: Hogrefe

[B51a] Yaniv I. Choshen-Hillel S. (2012). Exploiting the wisdom of others to make better decisions: suspending judgment reduces egocentrism and increases accuracy. J. Behav. Decis. Making 25, 427–434 10.1002/bdm.740

